# The mediating role of plasma glial fibrillary acidic protein in amyloid and tau pathology in Down's syndrome

**DOI:** 10.1002/alz.14359

**Published:** 2024-11-13

**Authors:** Anna H. Boerwinkle, Julie K. Wisch, Benjamin L. Handen, Elizabeth Head, Mark Mapstone, Michael S. Rafii, Sid E. O'Bryant, Sharon J. Krinsky‐McHale, Florence Lai, H. Diana Rosas, Shahid Zaman, Ira T. Lott, Dana Tudorascu, Matthew Zammit, Adam M. Brickman, Joseph H. Lee, Beau M. Ances, the Alzheimer's Biomarker Consortium‐Down Syndrome

**Affiliations:** ^1^ McGovern Medical School University of Texas in Houston Houston Texas USA; ^2^ Department of Neurology Washington University in St. Louis St. Louis Missouri USA; ^3^ Department of Psychiatry University of Pittsburgh Pittsburgh Pennsylvania USA; ^4^ Department of Pathology, Gillespie Neuroscience Research Facility University of California ‐ Irvine Irvine California USA; ^5^ Department of Neurology University of California Irvine School of Medicine Orange California USA; ^6^ Alzheimer's Therapeutic Research Institute Keck School of Medicine of USC Los Angeles California USA; ^7^ Institute for Translational Research Department of Pharmacology and Neuroscience University of North Texas Health Science Center Fort Worth Texas USA; ^8^ Department of Psychology New York State Institute for Basic Research in Developmental Disabilities Staten Island New York USA; ^9^ Department of Radiology, Harvard Medical School Massachusetts General Hospital Boston Massachusetts USA; ^10^ Department of Neurology, Harvard Medical School Massachusetts General Hospital Boston Massachusetts USA; ^11^ Cambridge Intellectual and Developmental Disabilities Research Group University of Cambridge Cambridge UK; ^12^ Department of Pediatrics University of California Irvine School of Medicine Orange California USA; ^13^ Department of Medical Physics and Psychiatry University of Wisconsin Madison Madison Wisconsin USA; ^14^ Department of Neurology Columbia University Irving Medical Center New York New York USA; ^15^ Department of Epidemiology Columbia University Irving Medical Center New York New York USA

**Keywords:** Alzheimer's disease, biomarkers, Down's syndrome, plasma

## Abstract

**INTRODUCTION:**

Development of Alzheimer's disease (AD) pathology in Down's syndrome (DS) occurs within a compressed timeline compared to sporadic or other genetic forms of AD.

**METHODS:**

Plasma glial fibrillary acidic protein (GFAP) and plasma pTau‐217 levels were compared by AD pathophysiology (amyloid (A+) and tau (T+) positron emission tomography [PET]) in persons with DS (*N* = 348) and sibling controls (*N* = 42). Plasma GFAP, plasma pTau‐217, amyloid‐PET, and tau‐PET levels were compared with regard to estimated years to onset of clinical symptoms (52.5 years old). We evaluated if plasma GFAP mediated the relationship between amyloid PET and plasma pTau‐217 or tau PET.

**RESULTS:**

Plasma GFAP, a measure of astrogliosis, was elevated in A+/T‐ and A+/T+ individuals with DS. Plasma pTau‐217 was elevated in A+/T+ individuals with DS. GFAP partially mediated the relationship between amyloid‐PET and tau‐PET (15.3%) and amyloid‐PET and plasma pTau‐217 (42.1%).

**DISCUSSION:**

Astrogliosis is a key component in the advancement of preclinical AD pathophysiology in DS.

**Highlights:**

- Amyloid may be a necessary precursor for stimulating astrocytes.
- Astrogliosis may play a key role in modifications to tau phosphorylation.
- Targeting neuroinflammation may only aid amyloid positive individuals.
- Alzheimer's disease timecourse is compressed in individuals with Down's syndrome.

## 1 INTRODUCTION

Down's syndrome (DS) is defined by full or partial triplication of chromosome 21.^1^ The amyloid precursor protein (*APP*) gene, an important component in the development of Alzheimer's disease (AD), is located on chromosome 21.^1^ Individuals with DS produce increased levels of amyloid‐β protein (Aβ) and typically develop dementia before 60 years of age.^2^ Over the past few decades, there has been a dramatic increase in the life expectancy of individuals with DS^3^ due to medical interventions. As result, a rapidly growing population of aging adults with DS will develop AD.

Important progress has been made in understanding the pathological and cognitive progression of AD in persons with DS. Similar to other forms of AD, amyloid aggregation occurs in persons with DS more than a decade before cognitive symptoms emerge.^4^ In sporadic and other genetic forms of AD, amyloid changes are followed by the accumulation of tau tangles a decade later.^5^, ^6^ In contrast, recent studies have shown that tau changes in DS occur only 2–5 years after amyloid accumulation.^5^, ^6^, ^7^ These results suggest a compressed timeline for developing AD in persons with DS; however, the etiology of this acceleration is not known.

Inflammation may play a key role in the progression of AD pathology.^8^ Plasma‐based measurement of glial fibrillary acidic protein (GFAP) is a reliable and less invasive measure of neuroinflammation compared to positron emission tomography (PET) imaging and cerebrospinal fluid (CSF) measures. GFAP is an intermediate filament protein found in astrocytes, elevated in the setting of astrogliosis, and an early biomarker of neuroinflammation in AD.^9^ Studies in DS, sporadic, and autosomal‐dominant AD have shown elevated plasma GFAP levels in amyloid‐positive individuals prior to significant tau accumulation and cognitive impairment.^10^, ^11^, ^12^ Neuroinflammation may mediate the development of tau pathology that occurs after the accumulation of amyloid.

Inflammation may be particularly important in DS. In addition to the APP gene, several inflammation‐related genes are located on chromosome 21.^1^ Prior studies in persons with DS have reported elevated plasma and CSF measures of inflammation, such as visinin‐like protein 1 (VILIP‐1), YKL‐40 (also known as Chitinase 3‐like 1), and tumor necrosis factor alpha (TNF‐α).^13^, ^14^ We sought to understand how elevated inflammation may influence AD biomarker progression in DS. We first determined when plasma GFAP elevation occurs in relation to amyloid and tau changes (as measured by PET imaging and plasma pTau‐217). We then performed mediation analyses to determine whether plasma GFAP mediates the relationship between amyloid PET and pTau‐217/PET tau burden in persons with DS. Understanding the role of inflammation in the compressed timeline of AD progression in DS may have important implications for clinical trials for AD in persons with DS.

## 2 METHODS

The Alzheimer's Biomarker Consortium – Down Syndrome (ABC‐DS) is a multi‐site study that enrolls adults with DS (≥25 years) and sibling‐controls and collects longitudinal clinical, imaging, and fluid biomarker data. Informed consent, or assent when appropriate, is obtained from all participants and from their legally authorized representative when necessary. Participants with DS and sibling controls were included if they had plasma GFAP, plasma pTau‐217, amyloid PET, and/or tau PET data available in the third data freeze (May 2023). While plasma was collected at baseline, some amyloid PET (*N* = 7) and tau PET (*N* = 21) was collected at the 18 month follow‐up visit (Tables S1 and S2). Abbreviated methods are provided with additional information in the supplement. This study was approved by the Institutional Review Board (IRB) at each site.

### 2.1 Cognitive assessment

ABC‐DS uses consensus diagnosis to determine cognitive status of participants with DS.^15^ Participants with DS were given a consensus diagnosis of either “cognitively stable,” “mild cognitive impairment,” “dementia,” or “no consensus” if an agreed consensus diagnosis was not reached. We considered participants with either mild cognitive impairment or dementia to be symptomatic, while participants evaluated as cognitively stable were considered asymptomatic.

### 2.2 Plasma collection and processing

Plasma GFAP was measured using Simoa kit (Quantrix, Lexington, MA) (Table S3). Plasma pTau‐217 was measured by an immunoassay on a Mesoscale Discovery platform (Table S4).^16^ Apolipoprotein E (*APOE)* genotype was determined from the blood samples using KASP genotyping assays (LGC Genomics, Beverly, MA). Individuals were categorized as *APOE* ε4 positive if they had at least one *ε4* allele.

### 2.3 MR and PET imaging

Amyloid PET imaging was collected in a subset of controls (*n* = 34) and participants with DS (*n* = 211) using either [^11^C]‐Pittsburgh Compound B (PiB) or [^18^F]‐AV45 (Florbetapir) (Table S5). Another subset of controls (*n* = 37) and persons with DS (*n* = 158) underwent tau PET imaging using [^18^F]‐AV1451 (Flortaucipir) (Table S6). T1‐weighted MR scans were collected on a 3‐Tesla scanner for all participants with PET imaging.

PET images were processed and aligned to FreeSurfer MR segmentations (v5.3) using an established processing pipeline (PET Unified Pipeline; https://github.com/ysu001/PUP).^17^ Because it is known that PET values are sensitive to image segmentation techniques,^18^ a sensitivity analysis was performed relying on a second processing pipeline that used Automated Anatomical Labeling (AAL).^19^ For both methods, regional standard uptake value ratios (SUVRs) were calculated using the cerebellar cortex as the reference region. Global amyloid burden was standardized across tracers using the Centiloid scale.^20^ A comparison of the two methods is available in Figure S1.

A tau PET summary region was calculated based on the average SUVRs from regions of interest that reflect Braak Stages I/III/IV.^19^, ^20^ Amyloid‐positivity was defined as a Centiloid > 18 and tau‐positivity was defined as tau summary SUVR > 1.3.^21^, ^22^

**RESEARCH IN CONTEXT**

1. **Systematic review**: The authors reviewed the literature using traditional (e.g., PubMed) sources and meeting abstracts. Recent publications have identified associations between positron emission tomography (PET) markers of amyloid and tau with plasma markers of glial fibrillary acidic protein (GFAP) and pTau‐217 in various forms of Alzheimer disease (AD); however, no mediation analysis has been performed in Down's syndrome (DS) to investigate the role of astrogliosis in changes in tau phosphorylation and deposition.
2. **Interpretation**: Our findings indicate that astrocyte‐related changes may play a key role in progression to tau phosphorylation. The relationships that we observe in DS are consistent with published observations of an accelerated disease time course in DS AD compared to other forms of AD.
3. **Future directions**: Interventions that target pathological inflammation soon after amyloid accumulation should be evaluated as a means to slow tau aggregation within persons with DS.

### 2.4 Statistical analysis

Differences between individuals with DS and sibling controls in demographic characteristics were evaluated using χ^2^‐tests for categorical variables and Kruskal‐Wallis rank sum tests for continuous variables. Comparisons of plasma GFAP and pTau‐217 between controls and persons with DS grouped by biomarker status were performed using the Kruskal Wallis test followed by pairwise Wilcoxon rank sum tests, correcting for multiple comparisons with Bonferroni correction. To compare the timing of plasma GFAP, plasma pTau‐217, amyloid PET, and tau PET changes, biomarkers were evaluated relative to age. For continuity with prior work, we also compared it relative to estimated years to symptom onset (EYO).^4^, ^6^ EYO was calculated by subtracting participant age from an average age of symptom onset (AAO) in DS. We used 52.5 years as the AAO.^4^, ^6^ Generalized additive models with a cubic regression spline were fitted for each biomarker as the response variable and EYO as the independent variable. The timing of super‐threshold accumulation for each biomarker was estimated using a 10,000 iteration bootstrap. Finally, a mediation analysis was performed on all individuals with complete information (*N* = 130, Table S7) to assess whether plasma GFAP explained the relationship between amyloid and both tau PET and plasma pTau‐217. All analyses controlled for latency in days between measures, gender, and APOE ε4 status.

## 3 RESULTS

Persons with DS (*n* = 348) and sibling controls (*n* = 42) were included (Table 1). The hundred and thirty‐nine participants (302 with DS) had plasma biomarker measures, 245 (211 with DS) had amyloid PET, and 195 (158 with DS) had tau PET. Controls and participants with DS were similar in age, racial identity, and *APOE ε4* positivity status (Table 1). There were more females in the control group (*p < 0.001*).

**TABLE 1:** Participant demographics

Parameter	Controls (*n* = 42)	Down syndrome (DS) (*n* = 348)	*p*‐Value
Age, years (mean [SD])	43.57 [12.5]	44.93 [9.7]	0.409
Female	33 (78.6%)	157 (45.1%)	**0.003**
Race			0.781
White	42 (100%)	334 (96%)	
Black or African American	0	4 (1.2%)	
Asian	0	5 (1.4%)	
Multi/other	0	5 (1.4%)	
Apolipoprotein E ε4‐positive	11 (26.2%)	81 (23.4%)	0.835
Consensus diagnosis			**–**
Asymptomatic	–	251 (72.1%)	
Mild cognitive impairment	–	41 (11.8%)	
Dementia	–	41 (11.8%)	
No consensus	–	15 (4.3%)	
Amyloid status (%)			–
Control	42 (100%)	–	
Unknown status	–	137 (39.4%)	
Amyloid negative	–	118 (33.9%)	
Amyloid positive	–	93 (26.7%)	
Tau status (%)			**–**
Control	42 (100%)	–	
Unknown status	–	190 (54.6%)	
Tau negative	–	119 (34.2%)	
Tau positive	–	39 (11.2%)	
Down's syndrome type			**—**
Full trisomy 21	**–**	303 (89.9%)	
Translocation	**–**	19 (5.6%)	
Mosaicism	**–**	15 (4.5%)	

**FIGURE 1:**
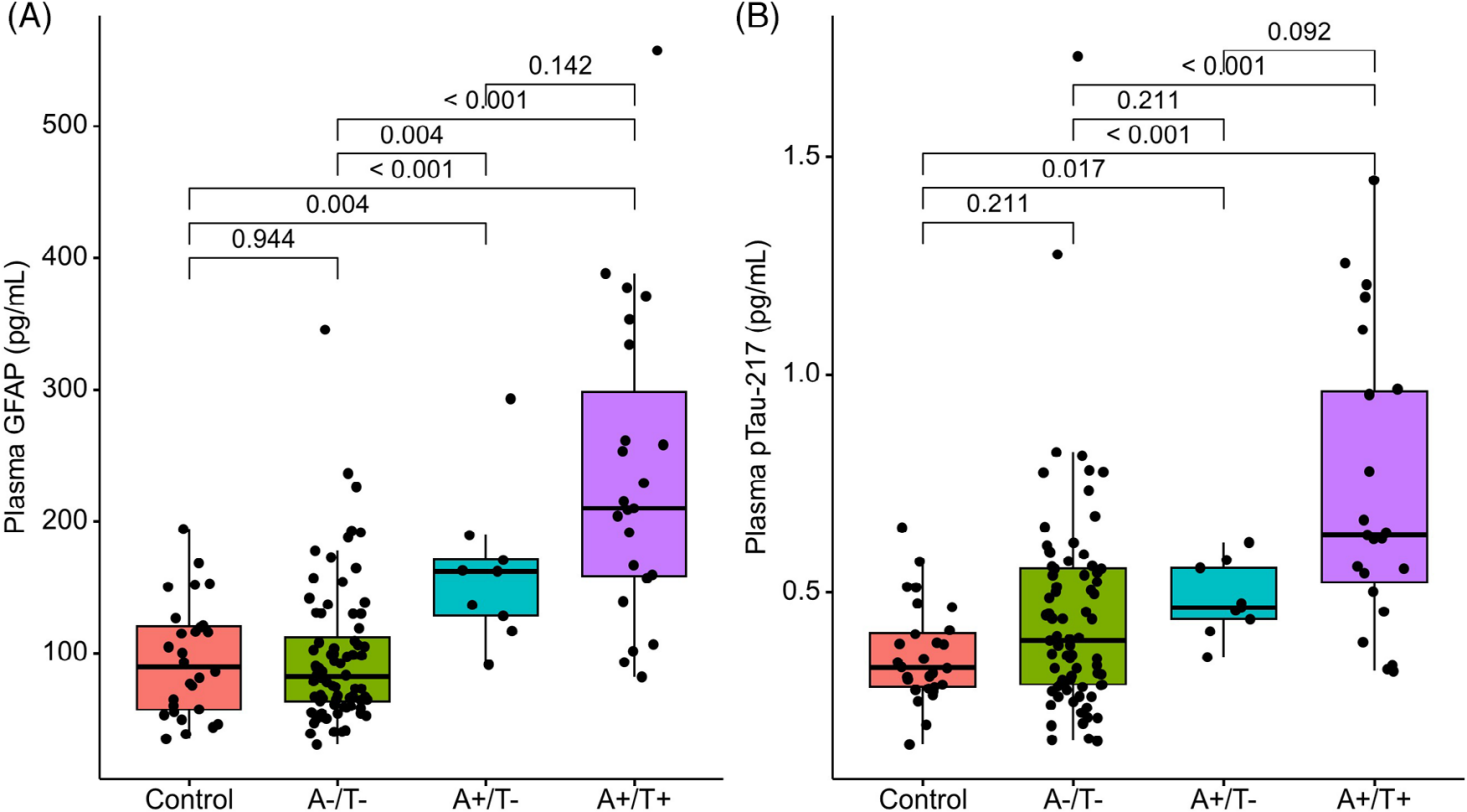
Comparison of plasma glial fibrillary acidic protein (GFAP) and ptau 217 as a function of Alzheimer's disease (AD) pathological groups (A‐/T‐: amyloid negative/tau negative; A+/T‐: amyloid positive/tau negative; A+/T+: amyloid positive/tau positive). (A) Plasma GFAP increased in a stair step manner, where plasma GFAP levels for A+/T‐ were significantly higher than sibling controls and A‐/T‐ participants. A+/T+ participants had the highest plasma GFAP, although it did not significantly differ from A+/T‐ participants. (B) Plasma pTau‐217 followed a similar stair step pattern where plasma pTau‐217 was significantly elevated for individuals who were A+/T‐ over controls, but not A‐/T‐ participants, suggesting that it changes later than plasma GFAP

Plasma GFAP and plasma pTau‐217 were compared by amyloid and tau PET‐positivity (Table S5). Two DS participants were A‐/T+ and excluded from analyses. Plasma GFAP did not differ between sibling controls and persons with DS who were A‐/T‐ (*p = 0.944*) (Figure 1A). Persons with DS who were A+/T‐ had significantly elevated plasma GFAP compared to A‐/T‐ *(64.343, 95% CI: 32.394, 96.181 pg/mL*) and sibling controls *(61.780, 95% CI: 23.434, 99.126 pg/mL*, *p* = 0.001). Persons with DS who were A+/T+ had higher GFAP compared to A‐T‐ (*125.200, 95% CI: 87.752, 161.180 pg/mL*, *p* < 0.001) but not A+/T‐ (*63.573, 95% CI: ‐5.210, 133.198 pg/mL, p = 0.142*). Plasma pTau‐217 was elevated in both A+/T‐ and A+/T+ individuals over sibling controls (*0.137, 95%CI: 0.060, 0.201 pg/mL, p < 0.001; 0.309, 95%CI: 0.200, 0.488 pg/mL, p = 0.017 respectively*). Plasma pTau‐217 was also elevated for A+/T‐ individuals over A‐/T‐ individuals with DS (*0.267, 95%CI: 0.136, 0.391, p < 0.001*) (Figure 1B). These results were robust to segmentation method (Figure S2).

**FIGURE 2:**
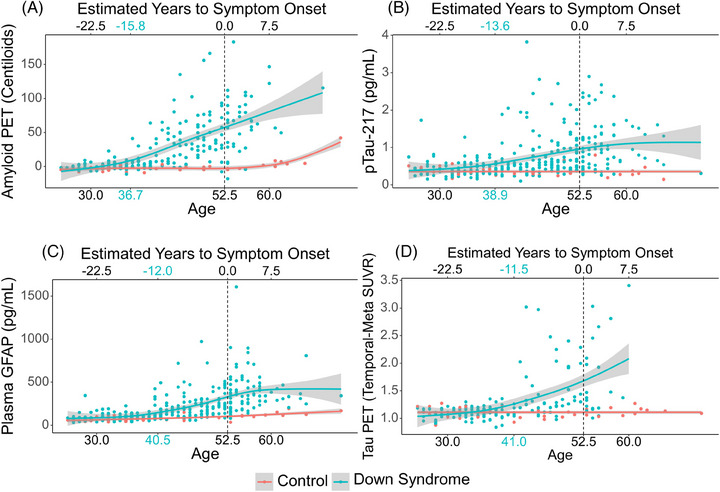
Comparison of Alzheimer's disease (AD) biomarkers in persons with Down's syndrome (DS) as a function of age/estimated years to onset of symptoms. (A) Persons with DS have significantly higher levels of amyloid positron emission tomography (PET) compared to controls at 36.7 years of age. (B) Persons with DS have significantly higher levels of plasma pTau‐217 compared to controls at 38.9 years of age. (C) Persons with DS have significantly higher levels of plasma glial fibrillary acidic protein (GFAP) compared to controls at 40.5 years of age. (D) Persons with DS have significantly higher levels of tau PET compared to controls at 41 years of age.

Amyloid PET increased earliest, with elevations seen in persons with DS compared to sibling controls at age 36.7 (Figure 2A). Plasma pTau‐217 was significantly elevated at 38.9 years for persons with DS compared to sibling controls (Figure 2B). The timing of changes in amyloid PET and plasma pTau‐217 was not significantly different (*p = 0.163*). These biomarker changes were followed by elevated levels of plasma GFAP and tau PET at 40.5 and 41 years of age, respectively, for DS participants compared to sibling controls (Figure 2C and 2D). Elevations in plasma GFAP and tau PET occurred significantly later than amyloid PET (*p = 0.017 and 0.016* respectively) but not plasma pTau‐217. Results for the alternative segmentation method were identical and thus not included in the Supplement.

When mediation analyses were performed in the set of participants with DS with full data (*N* = 130), plasma GFAP mediated 15.3% of the relationship between amyloid PET and tau PET (*p = 0.038;* Table S8), and 42.1% of the relationship between amyloid PET and plasma pTau‐217 (*p < 0.001;* Table S10). These results persisted whether or not APOEε4 status was included in the model. Results for the alternative segmentation were nearly identical (Tables S9 and S11).

## 4 DISCUSSION

Changes in neuro‐inflammation, as measured by GFAP, were quantified and placed in the context of other pathological biomarkers in persons with DS. Plasma GFAP increased after observable increases in amyloid‐PET and more proximally to observed changes in tau‐PET. Plasma GFAP was similar for controls and A‐/T‐ persons with DS, but was elevated in A+/T‐ and even higher for A+/T+ groups. Plasma pTau‐217 was elevated only in the A+/T+ group. The temporal placement of GFAP elevation occurring only after A+ but prior to T+ raises the notion that GFAP is an intermediator of AD pathogenesis. This effect of GFAP was seen for both amyloid‐PET and tau‐PET (15.3%) and amyloid‐PET and plasma pTau‐217 (42.1%).

Previous studies have suggested that inflammation‐related genes that reside on chromosome 21 may lead to elevation in neuroinflammatory pathways in persons with DS.^23^ The absence of elevated plasma GFAP in A‐/T‐ individuals with DS suggests that amyloid is a precursor for stimulating astrocytes, rather than a chronic state of increased neuroinflammation due to developmental differences. Thus, any intervention targeted at neuroinflammation may have the greatest effect in those individuals who are amyloid positive.

With regard to time, plasma GFAP was elevated after amyloid accumulation but before tau deposition when placed in the context of EYO, consistent with.^11^ This is consistent with work in sporadic AD that suggests GFAP increases in correspondence with amyloid pathology rather than tau pathology.^12^, ^24^ Increases in GFAP occurred less than 4 years after amyloid PET elevation. This is in contrast to a previous study in autosomal dominant AD where elevations in GFAP occurred 8 years after an increase in amyloid.^10^ Our findings support an accelerated progression of AD pathology in DS compared to other forms of AD.^4^, ^6^, ^7^

The mediation analysis provides potential key insights into the mechanisms involved. Reactive astrocytes release growth factors and neurotrophic factors that can modulate intracellular signaling pathways and increase tau phosphorylation.^25^ Increases in GFAP explained a large proportion of the relationship between amyloid PET and pTau‐217 (42%), suggesting astrocyte‐related changes may play a key role in progression to tau phosphorylation. Excessive phosphorylation of tau increases the probability of tau aggregating into the hallmarks of AD pathology: insoluble paired helical filaments and neurofibrillary tangles.^26^ GFAP explained a smaller but still significant proportion of the relationship between amyloid PET and tau PET, suggesting that astrocyte response as measured by GFAP may be more upstream of tau tangle deposition, impacting phosphorylation of tau. Although longitudinal tau measurement is not yet available in ABC‐DS, one study in sporadic AD using longitudinal tau PET identified that individuals with elevated plasma GFAP had an accelerated rate of tau accumulation over time.^25^ Future analyses that include longitudinal tau PET are needed to fully describe the inflammatory cascade involved in the development of AD pathology in DS.

This study was limited to cross‐sectional data. Longitudinal data that are actively being acquired by ABC‐DS will greatly enhance our understanding of biomarker change over time. Another limitation is the relatively few persons with DS who had cognitive impairment (22%). Additional studies that include participants with more advanced disease are needed. The current study focused on early changes seen with preclinical AD. Although GFAP obtained via plasma is known to have better correspondence with preclinical AD pathology than CSF,^9^, ^12^, ^24^ additional PET measures of neuroinflammation should be considered. This study is also limited by ethnoracial diversity. Future studies with increased enrollment of non‐White individuals are necessary to ensure generalizability. Another potential future avenue for research is differences by biological sex. Current differences in proportion of female participants in controls as compared to individuals with DS make it possible that observed differences could be confounded by sex differences.

In conclusion, elevation in plasma GFAP occurred after changes in amyloid‐PET but prior to elevations in plasma pTau‐217 and tau‐PET, with all biomarkers changing within a narrow (∼5 year) window. These results are consistent with a compressed timeline of AD pathology in DS. Plasma GFAP was a significant partial mediator of both the relationship between amyloid‐PET and tau‐PET as well as amyloid‐PET and pTau‐217, suggesting that astrogliosis is a key step in AD development in DS. A combination of interventions that target pathological inflammation soon after amyloid accumulation may slow tau aggregation within persons with DS.

## CONFLICT OF INTEREST STATEMENT

B.L.H. has received research funding from Roche and Autism Speaks; receives royalties from Oxford University Press for book publications; and is the chair of the data safety and monitoring board for the Department of Defense‐funded study, “Comparative Effectiveness of EIBI and MABA”. B.T.C. receives research funding from the National Institutes of Health. E.H. receives research funding from the National Institutes of Health and the BrightFocus Foundation. F.L. is supported by grants from the National Institute on Aging. H.D.R. has received funding from the National Institutes of Health and is on the scientific advisory committee for the Hereditary Disease Foundation. J.H.L. has received research funding from the National Institutes of Health and the National Institute on Aging. B.M.A. receives research funding from the National Institutes of Health and has a patent (“Markers of Neurotoxicity in CAR T patients”). M.S.R. has received consulting fees from AC Immune, Embic, and Keystone Bio and has received research support from the National Institutes of Health, Avid, Baxter, Eisai, Elan, Genentech, Janssen, Lilly, Merck, and Roche. All other authors declare no competing interests.

## Supporting information



Supporting Information

Supporting Information

## Data Availability

The data used in this analysis are available on request, provided applications are approved by the ABC‐DS committee. The data request application is available at https://pitt.co1.qualtrics.com/jfe/form/SV_cu0pNCZZlrdSxUN

## References

[1] Mrak RE , Griffin WST . Trisomy 21 and the Brain. J Neuropathol Exp Neurol. 2004;63:679‐685. 15290893 10.1093/jnen/63.7.679 PMC3833615

[2] Fortea J , Zaman SH , Hartley S , Rafii MS , Head E , Carmona‐Iragui M . Alzheimer's disease associated with Down syndrome: a genetic form of dementia. Lancet Neurol. 2021;20:930‐942. 34687637 10.1016/S1474-4422(21)00245-3 PMC9387748

[3] de Graaf G , Buckley F , Dever J , Skotko BG . Estimation of live birth and population prevalence of Down syndrome in nine U.S. states. Am J Med Genet A. 2017;173:2710‐2719. 28816027 10.1002/ajmg.a.38402

[4] Boerwinkle AH , Gordon BA , Wisch J , et al. Comparison of amyloid burden in individuals with Down syndrome versus autosomal dominant Alzheimer's disease: a cross‐sectional study. Lancet Neurol. 2023;22:55‐65. 36517172 10.1016/S1474-4422(22)00408-2 PMC9979840

[5] Masters CL , Bateman R , Blennow K , Rowe CC , Sperling RA , Cummings JL . Alzheimer's Disease. Nat Rev Dis Primers. 2015;1:15056. 27188934 10.1038/nrdp.2015.56

[6] Wisch JK , Mckay NS , Boerwinkle AH , et al. Comparison of tau spread in people with Down syndrome versus autosomal dominant Alzheimer's disease: a cross‐sectional study. Lancet Neurol. 2024. 10.1016/S1474-4422(24)00084-X PMC11209765 38631766

[7] Zammit MD , Betthauser TJ , McVea AK , et al. Characterizing the emergence of amyloid and tau burden in Down syndrome. Alzheimer's & Dementia. 2023; doi:10.1002/alz.13444 PMC10843570 37641577

[8] Bieger A , Rocha A , Bellaver B , et al. Neuroinflammation biomarkers in the AT(N) framework across the Alzheimer's disease continuum. J Prev Alzheimers Dis. 2023;10:401‐417. doi:10.14283/jpad.2023.54 37357281

[9] Abdelhak A , Foschi M , Abu‐Rumeileh S , et al. Blood GFAP as an emerging biomarker in brain and spinal cord disorders. Nat Rev Neurol. 2022;18:158‐172. 35115728 10.1038/s41582-021-00616-3

[10] Chatterjee P , Vermunt L , Gordon BA , et al. Plasma glial fibrillary acidic protein in autosomal dominant Alzheimer's disease: associations with Aβ‐PET, neurodegeneration, and cognition. Alzheimer's & Dementia. 2023;19:2790‐2804. 10.1002/alz.12879 PMC10300233 36576155

[11] Montoliu‐Gaya L , Alcolea D , Ashton NJ , et al. Plasma and cerebrospinal fluid glial fibrillary acidic protein levels in adults with Down syndrome: a longitudinal cohort study. EBioMedicine. 2023;90:104547. 37002988 10.1016/j.ebiom.2023.104547 PMC10070083

[12] Benedet AL , Milà‐Alomà M , Vrillon A , et al. Differences between plasma and cerebrospinal fluid glial fibrillary acidic protein levels across the Alzheimer disease continuum. JAMA Neurol. 2021;78:1471. 34661615 10.1001/jamaneurol.2021.3671 PMC8524356

[13] Fagan AM , Henson RL , Li Y , et al. Comparison of CSF biomarkers in Down syndrome and autosomal dominant Alzheimer's disease: a cross‐sectional study. Lancet Neurol. 2021;20:615‐626. 34302786 10.1016/S1474-4422(21)00139-3 PMC8496347

[14] O'bryant SE , Zhang F , Johnson LA , et al. A precision medicine model for targeted NSAID therapy in Alzheimer's disease. J Alzheimers Dis. 2018;66:97‐104. 30198872 10.3233/JAD-180619 PMC6428063

[15] Hartley SL , Handen BL , Tudorascu D , et al. Role of tau deposition in early cognitive decline in Down syndrome. Alzheimers Dement (Amst). 2022;14:e12256. 35386473 10.1002/dad2.12256 PMC8976157

[16] Palmqvist S , Janelidze S , Quiroz YT , et al. Discriminative Accuracy of Plasma Phospho‐tau217 for Alzheimer Disease vs Other Neurodegenerative Disorders. JAMA. 2020;324:772. 32722745 10.1001/jama.2020.12134 PMC7388060

[17] Su Yi , D'angelo GM , Vlassenko AG , et al. Quantitative analysis of PiB‐PET with FreeSurfer ROIs. PLoS One. 2013;8:e73377. 24223109 10.1371/journal.pone.0073377 PMC3819320

[18] Tudorascu DL , Minhas DS , Lao PJ , et al. The use of Centiloids for applying [^11^C]PiB classification cutoffs across region‐of‐interest delineation methods. Alzheimers Dement (Amst). 2018;10:332‐339. 30014032 10.1016/j.dadm.2018.03.006 PMC6024172

[19] Zammit MD , Laymon CM , Betthauser TJ , et al. Amyloid accumulation in Down syndrome measured with amyloid load. Alzheimers Dement (Amst). 2020;12:e12020. 32435686 10.1002/dad2.12020 PMC7233422

[20] Klunk WE , Koeppe RA , Price JC , et al. The Centiloid project: standardizing quantitative amyloid plaque estimation by PET. Alzheimers Dement. 2015;11:1‐15.e4. 25443857 10.1016/j.jalz.2014.07.003 PMC4300247

[21] Zammit MD , Tudorascu DL , Laymon CM , et al. PET measurement of longitudinal amyloid load identifies the earliest stages of amyloid‐beta accumulation during Alzheimer's disease progression in Down syndrome. Neuroimage. 2021;228:117728. 33421595 10.1016/j.neuroimage.2021.117728 PMC7953340

[22] Janelidze S , Christian BT , Price J , et al. Detection of Brain Tau Pathology in Down Syndrome Using Plasma Biomarkers. JAMA Neurol. 2022;79:797. 35789365 10.1001/jamaneurol.2022.1740 PMC9257682

[23] Wilcock DM , Griffin WST . Down's syndrome, neuroinflammation, and Alzheimer neuropathogenesis. J Neuroinflammation. 2013;10:864. 10.1186/1742-2094-10-84 PMC3750399 23866266

[24] Pereira JB , Janelidze S , Smith R , et al. Plasma GFAP is an early marker of amyloid‐β but not tau pathology in Alzheimer's disease. Brain. 2021;144:3505‐3516. 34259835 10.1093/brain/awab223 PMC8677538

[25] Bellaver B , Povala G , Ferreira PCL , et al. Astrocyte reactivity influences amyloid‐β effects on tau pathology in preclinical Alzheimer's disease. Nat Med. 2023;29:1775‐1781. 37248300 10.1038/s41591-023-02380-x PMC10353939

[26] Barthélemy NR , Li Y , Joseph‐Mathurin N , Gordon BA , et al. A soluble phosphorylated tau signature links tau, amyloid and the evolution of stages of dominantly inherited Alzheimer's disease. Nat Med. 2020;26:398‐407. 32161412 10.1038/s41591-020-0781-z PMC7309367

