# Supplementary material for: The mediating role of plasma glial fibrillary acidic protein in amyloid and tau pathology in Down's syndrome
Source: Alzheimers Dement. 2024 Nov 13;21(1):e14359. doi: 10.1002/alz.14359 (PMC11782213; doi:10.1002/alz.14359)
Supplement: Supplementary file 1 — Supporting Information [file ALZ-21-e14359-s001.docx]

**METHODS SUPPLEMENT**

*Cognitive Assessment – Consensus Diagnosis*

At each visit, participants with DS undergo a series of neurocognitive evaluations specifically designed for adults with DS or assessments for early cognitive changes and dementia in DS. Participants with DS are given a clinical dementia status by a committee of at least three members with clinical training or extensive experience in evaluating dementia in persons with DS. This committee considers these evaluations and a number of other variables, including medical and psychiatric history and interviews with caregivers or other informants, but is blind to biomarker measures, APOE genotype, imaging findings, and supplemental measures of cognitive or functional status. Consistent with the recommendations of the AAMR-IASSID Working Group for the Establishment of Criteria for the Diagnosis of Dementia in Individuals with Developmental Disability, participants with DS are given a consensus diagnosis of either “cognitively stable”, “mild cognitive impairment”, “dementia”, or “no consensus” if an agreed consensus diagnosis cannot be reached ^1^.

*Plasma Collection and Processing*

Blood was collected from participants in 10 mL EDTA-plasma tubes (Vacutainer K2EDTA tube, BD Diagnostics). Blood collection was not necessarily administered under a fasting state or at a specific time of day. After collection, tubes were gently inverted 8 – 10 times, placed on wet ice, and then centrifuged. Following centrifugation (10 minutes, 4 ^o^C), plasma were transferred into polypropylene tubes, mixed, and aliquotted for storage at -80^o^C within 180 minutes of collection at local ABC-DS sites. Vials were then shipped from local sites to the National Cell Repository for Alzheimer’s Disease at Indiana University and continued to be stored at -80^o^C ^2^.

*MR and PET Imaging*

Amyloid PET imaging was collected in a subset of control (n = 34) and participants with DS (n = 211) using either [^11^C]-Pittsburgh Compound B (PiB) or [^18^F]-AV45 (Florbetapir) ^1^. A target dose of 15mCi of [^11^C]-PiB or 10mCi of [^18^F]-Florbetapir was administered via intravenous bolus injection. Amyloid PET scans were acquired 50-70 minutes (4 x 5 minute frames) post-injection and were corrected for motion between frames. A subset of controls (n = 37) and participants with DS (n = 158) underwent tau PET imaging using the tracer [^18^F]-AV1451 (Flortaucipir). A target dose of 10mCi of [^18^F]-Flortaucipir was administered via intravenous bolus injection. Tau PET scans were acquired 80-100 minutes (4 x 5 minute frames) post-injection and were corrected for motion between frames. A T1-weighted MR scan was collected on a 3-Tesla scanner for all participants with PET imaging.

To obtain PET tracer uptake measures, MR scans were first processed with FreeSurfer 5.3 using consistent quality control procedures to obtain regional segmentations. All PET images were then processed and aligned to these FreeSurfer MR segmentations using an established processing pipeline (PET Unified Pipeline; <https://github.com/ysu001/PUP>). PET images were processed as static images. Using FreeSurfer-defined regions of interest (ROIs), regional standard uptake value ratios (SUVRs) were calculated using the cerebellar cortex as the reference region. Global amyloid burden was harmonized across tracers using the Centiloid scale ^3^ (Equations 1 & 2).

$Centiloid=45*{SUVR}_{PiB,50-70min}-47.5$ (1)

$Centiloid=$53.6 * ${SUVR}_{AV45,50-70min}-43.2$ (2)

An additional PET processing pipeline exists for ABCDS, where processed values are currently available for download on LONI. This method has previously been described ^4^. Individual PET frames are summed to create a static image and spatially normalized to the Montreal Neurological Institute 152 space (MNI) using SPM12 with a Down Syndrome specific template^5^. PET image registration was inspected relative to corresponding MRI prior to spatial normalization. Using Automated Anatomical Labeling (AAL) atlas regions 91 – 108, SUVRs were calculated using the cerebellar cortex as the reference region. Global amyloid burden was harmonized using Equations 1 and 2.

A summary region for tau PET was calculated based on the average of the SUVRs in ROIs in which significant early stage tau accumulation is measured and approximate Braak stages I/III/IV: entorhinal cortex, inferior temporal cortex, middle temporal cortex, fusiform cortex, parahippocampal cortex, and amygdala. These regions were determined in a previous PET imaging study of tau spread and has been used in prior studies to define tau-positivity in Down syndrome ^6,7^. To categorize participants with DS as amyloid- and tau-positive, we used a threshold of Centiloid > 18 and tau summary SUVR > 1.3 ^7,8^.

**REFERENCES**

1. Handen, B. L. *et al.* The Alzheimer’s Biomarker Consortium‐Down Syndrome: Rationale and methodology. *Alzheimer’s & Dementia: Diagnosis, Assessment & Disease Monitoring* **12**, (2020).

2. Mapstone, M. *et al.* Metabolic correlates of prevalent mild cognitive impairment and Alzheimer’s disease in adults with Down syndrome. *Alzheimer’s & Dementia: Diagnosis, Assessment & Disease Monitoring* **12**, (2020).

3. Klunk, W. E. *et al.* The Centiloid project: Standardizing quantitative amyloid plaque estimation by PET. *Alzheimer’s and Dementia* **11**, 1-15.e4 (2015).

4. Zammit, M. D. *et al.* Amyloid accumulation in Down syndrome measured with amyloid load. *Alzheimer’s & Dementia: Diagnosis, Assessment & Disease Monitoring* **12**, (2020).

5. Lao, P. J. *et al.* Imaging neurodegeneration in Down syndrome: brain templates for amyloid burden and tissue segmentation. *Brain Imaging Behav* **13**, 345–353 (2019).

6. Cho, H. *et al.* In vivo cortical spreading pattern of tau and amyloid in the Alzheimer disease spectrum. *Ann Neurol* **80**, 247–258 (2016).

7. Janelidze, S. *et al.* Detection of Brain Tau Pathology in Down Syndrome Using Plasma Biomarkers. *JAMA Neurol* **79**, 797 (2022).

8. Zammit, M. D. *et al.* PET measurement of longitudinal amyloid load identifies the earliest stages of amyloid-beta accumulation during Alzheimer’s disease progression in Down syndrome. *Neuroimage* **228**, (2021).

**SUPPLEMENTAL TABLES AND FIGURES**

**
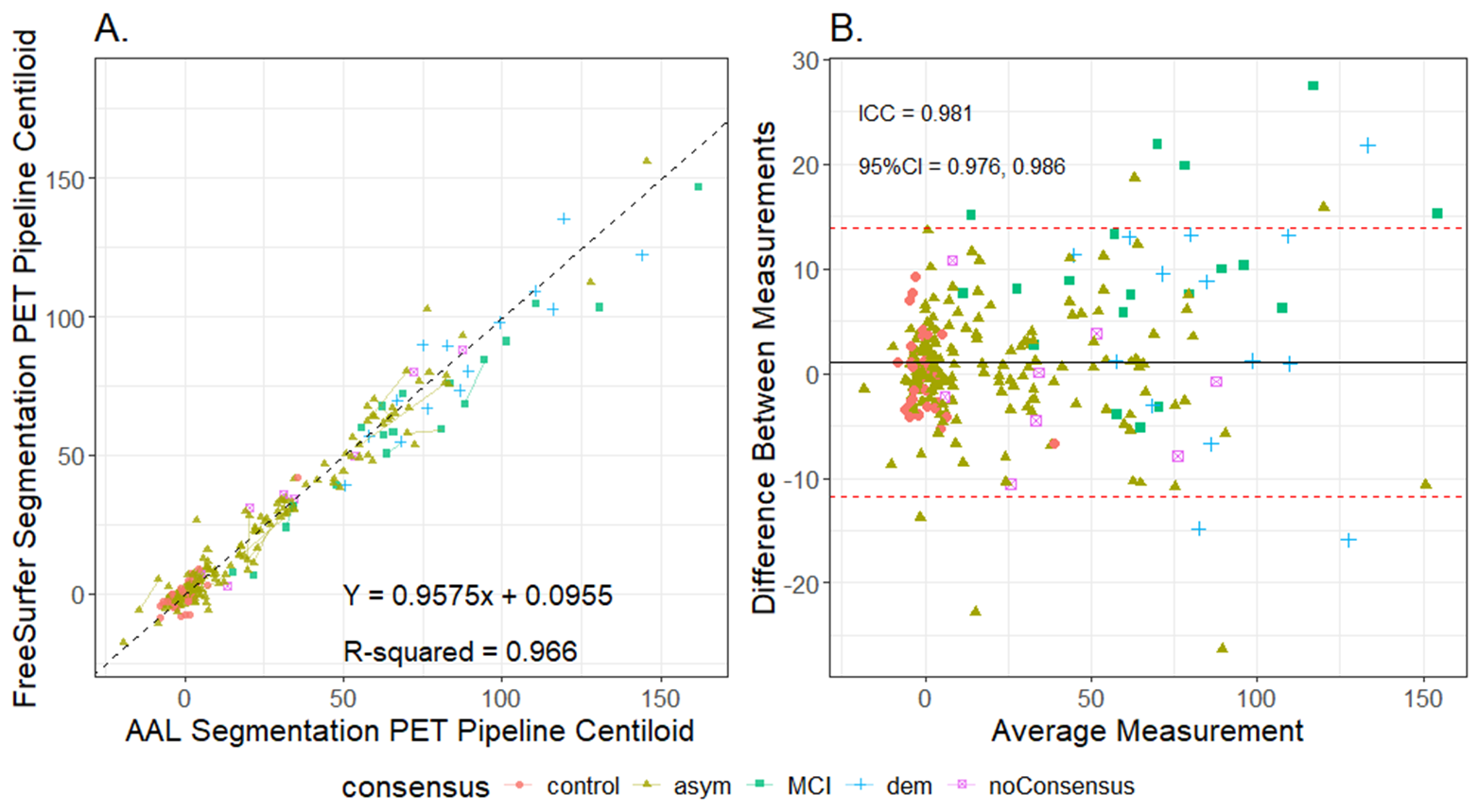
**

**Supplemental Figure 1. Comparison between Amyloid PET Processing pipelines.** (A.) Centiloid values between the two considered PET processing pipelines are highly similar as assessed by R^2^ value. (B.) Bland Altman plot reveals very high intra-class correlation (0.98) although some systematic bias exists for individuals with the highest levels of amyloid accumulation.

**
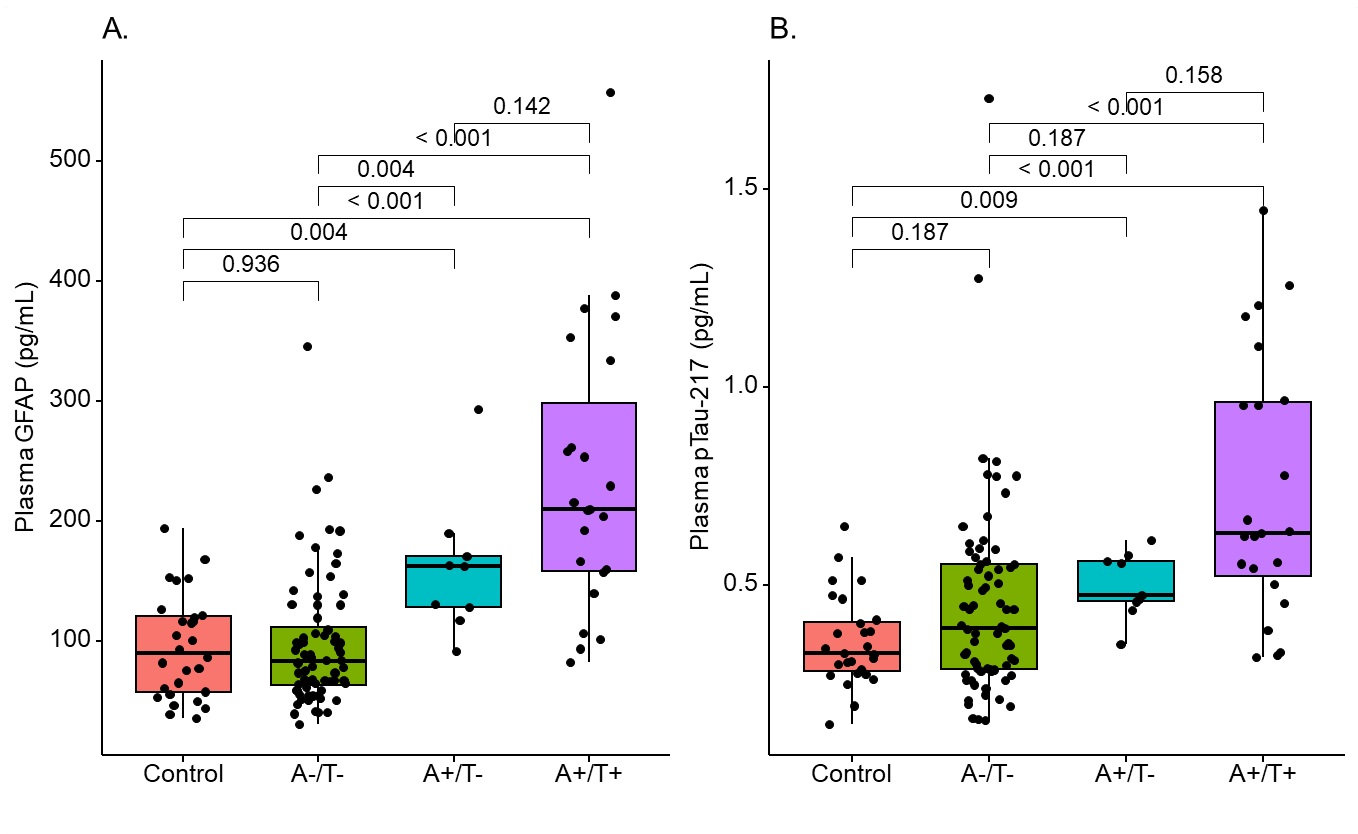
**

**Supplemental Figure 2. Comparison of plasma GFAP and ptau 217 as a function of AD Pathological Groups** (A-/T-: Amyloid Negative/Tau Negative; A+/T-: Amyloid Positive/Tau Negative; A+/T+: Amyloid Positive/Tau Positive). These results rely on amyloid PET classification based on segmentation via AAL instead of Freesurfer (in main text). They do not materially differ. (A) Plasma GFAP increased in a stair step manner, where plasma GFAP levels for A+/T- were significantly higher than sibling controls and A-/T- participants. A+/T+ participants had the highest plasma GFAP, although it did not significantly differ from A+/T- participants.. (B) Plasma pTau-217 followed a similar stair step pattern where plasma pTau-217 was significantly elevated for individuals who were A+/T- over controls, but not A-/T- participants, suggesting that it changes later than plasma GFAP.

**Supplemental Table 1.** Demographics of participants with amyloid PET, stratified by amyloid PET scan availability

|  | Controls, PET at Baseline  (n = 33) | Controls, PET at Followup  (n = 1) | DS, PET at Baseline  (n = 205) | DS, PET at Followup  (n = 6) | p-value |
| --- | --- | --- | --- | --- | --- |
| **Age, years (mean [SD])** | 44.06 [13.1] | 33.00 [NA] | 42.09 [9.1] | 55.00 [9.76] | 0.486 |
| **Female** | 27 (81.8%) | 1 (100%) | 90 (43.9%) | 5 (83.3%) | **< 0.001** |
| **Race** |  |  |  |  | 1 |
| White | 33 (100%) | 1 (100%) | 197 (96.1%) | 6 (100%) |  |
| Black or African American | -- | -- | 2 (1.0%) | -- |  |
| Asian | -- | -- | 3 (1.5%) | -- |  |
| Multi/Other | -- | -- | 3 (1.5%) | -- |  |
| **APOE ε4-positive** | 9 (27.3%) | 0 (0.0%) | 41 (19.5%) | 0 (0.0%) | 0.431 |
| **Consensus diagnosis** |  |  |  |  | **< 0.001** |
| Asymptomatic | -- | -- | 165 (80.5%) | 3 (50.0%) |  |
| Mild cognitive impairment | -- | -- | 16 (7.8%) | 1 (16.7%) |  |
| Symptomatic | -- | -- | 15 (7.3%) | 2 (33.3%) |  |
| No consensus | -- | -- | 9 (4.4%) | 0 (0.0%) |  |
| **Amyloid Classification** |  |  |  |  | **< 0.001** |
| Control | 33 (100%) | 1 (100%) | -- | -- |  |
| Amyloid Negative | -- | -- | 117 (57.1%) | 1 (16.7%) |  |
| Amyloid Positive | -- | -- | 88 (42.9%) | 5 (83.3%) |  |
| **Down syndrome type** |  |  |  |  | **< 0.001** |
| Full trisomy 21 | **--** | -- | 183 (91.0%) | 6 (100%) |  |
| Translocation | **--** | -- | 12 (6.0%) | -- |  |
| Mosaicism | **--** | -- | 6 (3.0%) | -- |  |

**Supplemental Table 2.** Demographics of participants with tau PET, stratified by tau PET scan availability

|  | Controls  (n = 37) | DS, PET at Baseline  (n = 137) | DS, PET at followup  (n = 21) | p-value |
| --- | --- | --- | --- | --- |
| **Age, years (mean [SD])** | 43.78 [13.0] | 38.60 [8.2] | 50.14 (5.4) | **< 0.001** |
| **Female** | 30 (81.1%) | 63 (46%) | 12 (57.1%) | **0.001** |
| **Race** |  |  |  | 0.295 |
| White | 37 (100%) | 134 (97.8%) | 20 (95.2%) |  |
| Black or African American | -- | -- | 1 (4.8%) |  |
| Asian | -- | 1 (0.7%) | -- |  |
| Multi/Other | -- | 2 (1.4%) | -- |  |
| **APOE ε4-positive** | 9 (24.3%) | 27 (20.0%) | 5 (23.8%) | 0.812 |
| **Consensus diagnosis** |  |  |  | **< 0.001** |
| Asymptomatic | -- | 120 (87.6%) | 17 (81.0%) |  |
| Mild cognitive impairment | -- | 6 (4.4%) | 2 (9.5%) |  |
| Symptomatic | -- | 4 (2.9%) | 2 (9.5%) |  |
| No consensus | -- | 7 (5.1%) | 0 |  |
| **Tau Classification** |  |  |  | **0.004** |
| Control | 37 (100%) | -- | -- |  |
| Tau Negative | -- | 109 (79.6%) | 10 (47.6%) |  |
| Tau Positive | -- | 28 (20.4%) | 11 (55.0%) |  |
| **Down syndrome type** |  |  |  | **< 0.001** |
| Full trisomy 21 | **--** | 143 (91.1%) | 189 (90.5%) |  |
| Translocation | **--** | 10 (6.4%) | 1 (4.8%) |  |
| Mosaicism | **--** | 4 (2.5%) | 1 (4.8%) |  |

**Supplemental Table 3.** Demographics of participants with plasma GFAP

|  | Controls  (n = 37) | Down syndrome (DS)  (n = 301) | p-value |
| --- | --- | --- | --- |
| **Age, years (mean [SD])** | 44.86 [12.7] | 45.17 [9.7] | 0.860 |
| **Female** | 30 (81.1%) | 139 (46.2%) | **< 0.001** |
| **Race** |  |  | 0.706 |
| White | 37 (100%) | 290 (96.3%) |  |
| Black or African American | 0 | 2 (0.7%) |  |
| Asian | 0 | 5 (1.7%) |  |
| Multi/Other | 0 | 4 (1.3%) |  |
| **APOE ε4-positive** | 9 (24.3%) | 72 (24%) | 1.000 |
| **Consensus diagnosis** |  |  | **--** |
| Asymptomatic | -- | 213 (70.8%) |  |
| Mild cognitive impairment | -- | 38 (12.6%) |  |
| Symptomatic | -- | 37 (12.3%) |  |
| No consensus | -- | 13 (4.3%) |  |
| **Amyloid / Tau Classification** |  |  | -- |
| Control | 37 (100%) | -- | -- |
| Unknown |  | 190 (63.1%) |  |
| A-/T- | -- | 76 (25.2%) |  |
| A+/T- | -- | 10 (3.3%) |  |
| A+/T+ | -- | 25 (8.3%) |  |
| **Down syndrome type** |  |  | **---** |
| Full trisomy 21 | **--** | 261 (89.7%) |  |
| Translocation | **--** | 16 (5.5%) |  |
| Mosaicism | **--** | 14 (4.8%) |  |

Abbreviations: APOE = apolipoprotein E; DS = Down syndrome; SD = standard deviation

**Supplemental Table 4.** Demographics of participants with plasma pTau-217

|  | Controls  (n = 37) | Down syndrome (DS)  (n = 302) | p-value |
| --- | --- | --- | --- |
| **Age, years (mean [SD])** | 44.86 [12.7] | 45.12 [9.7] | 0.885 |
| **Female** | 30 (81.1%) | 140 (46.4%) | **< 0.001** |
| **Race** |  |  | 0.707 |
| White | 37 (100%) | 291 (96.4%) |  |
| Black or African American | 0 | 2 (0.7%) |  |
| Asian | 0 | 5 (1.7%) |  |
| Multi/Other | 0 | 4 (1.3%) |  |
| **APOE ε4-positive** | 9 (24.3%) | 72 (23.9%) | 1.000 |
| **Consensus diagnosis** |  |  | **--** |
| Asymptomatic | -- | 213 (70.5%) |  |
| Mild cognitive impairment | -- | 38 (12.6%) |  |
| Symptomatic | -- | 37 (12.3%) |  |
| No consensus | -- | 14 (4.6%) |  |
| **Amyloid / Tau Classification** |  |  | -- |
| Control | 37 (100%) | -- |  |
| A-/T- | -- | 77 (25.5%) |  |
| A+/T- | -- | 10 (3.3%) |  |
| A+/T+ | -- | 25 (8.3%) |  |
| **Down syndrome type** |  |  | **---** |
| Full trisomy 21 | **--** | 262 (89.7%) |  |
| Translocation | **--** | 16 (5.5%) |  |
| Mosaicism | **--** | 14 (4.8%) |  |

Abbreviations: APOE = apolipoprotein E; DS = Down syndrome; SD = standard deviation

**Supplemental Table 5.** Demographics of participants with amyloid PET

|  | Controls  (n = 34) | Down syndrome (DS)  (n = 211) | p-value |
| --- | --- | --- | --- |
| **Age, years (mean [SD])** | 43.74 [13.0] | 42.45 [9.3] | 0.486 |
| **Female** | 28 (82.4%) | 95 (45.0%) | **< 0.001** |
| **Race** |  |  | 0.856 |
| White | 34 (100%) | 203 (96.2%) |  |
| Black or African American | 0 | 2 (0.9%) |  |
| Asian | 0 | 3 (1.4%) |  |
| Multi/Other | 0 | 3 (1.4%) |  |
| **APOE ε4-positive** | 9 (26.5%) | 41 (19.5%) | 0.483 |
| **Consensus diagnosis** |  |  | **--** |
| Asymptomatic | -- | 168 (79.6%) |  |
| Mild cognitive impairment | -- | 17 (8.1%) |  |
| Symptomatic | -- | 17 (8.1%) |  |
| No consensus | -- | 9 (4.3%) |  |
| **Amyloid / Tau Classification** |  |  | -- |
| Control | 34 (100%) | -- |  |
| A-/T- | -- | 94 (44.5%) |  |
| A+/T- | -- | 11 (5.2%) |  |
| A+/T+ | -- | 27 (12.8%) |  |
| **Down syndrome type** |  |  | **---** |
| Full trisomy 21 | **--** | 189 (91.3%) |  |
| Translocation | **--** | 12 (5.8%) |  |
| Mosaicism | **--** | 6 (2.9%) |  |

Abbreviations: APOE = apolipoprotein E; DS = Down syndrome; SD = standard deviation

**Supplemental Table 6.** Demographics of participant with tau PET

|  | Controls  (n = 37) | Down syndrome (DS)  (n = 158) | p-value |
| --- | --- | --- | --- |
| **Age, years (mean [SD])** | 43.78 [13.0] | 40.13 [9.3] | **0.040** |
| **Female** | 30 (81.1%) | 75 (47.5%) | **< 0.001** |
| **Race** |  |  | 0.916 |
| White | 37 (100%) | 154 (97.5%) |  |
| Black or African American | 0 | 1 (0.6%) |  |
| Asian | 0 | 1 (0.6%) |  |
| Multi/Other | 0 | 2 (1.3%) |  |
| **APOE ε4-positive** | 9 (24.3%) | 32 (20.5%) | 0.775 |
| **Consensus diagnosis** |  |  | **--** |
| Asymptomatic | -- | 137 (86.7%) |  |
| Mild cognitive impairment | -- | 8 (5.1%) |  |
| Symptomatic | -- | 6 (3.8%) |  |
| No consensus | -- | 7 (4.4%) |  |
| **Amyloid / Tau Classification** |  |  | -- |
| Control | 37 (100%) | -- |  |
| A-/T- | -- | 94 (59.5%) |  |
| A+/T- | -- | 11 (7.0%) |  |
| A+/T+ | -- | 27 17.1%) |  |
| **Down syndrome type** |  |  | **---** |
| Full trisomy 21 | **--** | 143 (91.1%) |  |
| Translocation | **--** | 10 (6.4%) |  |
| Mosaicism | **--** | 4 (2.5%) |  |

Abbreviations: APOE = apolipoprotein E; DS = Down syndrome; SD = standard deviation

**Supplemental Table 7.** Demographics of participant with amyloid PET, tau PET, plasma GFAP, and plasma pTau-217

|  | Controls  (n = 29) | Down syndrome (DS)  (n = 130) | p-value |
| --- | --- | --- | --- |
| **Age, years (mean [SD])** | 44.83 [13.5] | 40.72 [8.7] | **0.042** |
| **Female** | 25 (86.2%) | 68 (52.3%) | **0.002** |
| **Race** |  |  | 0.877 |
| White | 29 (100%) | 127 (97.7%) |  |
| Black or African American | 0 | 1 (0.8%) |  |
| Asian | 0 | 1 (0.8%) |  |
| Multi/Other | 0 | 1 (0.8%) |  |
| **---** | 7 (24.1%) | 25 (19.4%) | 0.749 |
| **Consensus diagnosis** |  |  | **--** |
| Asymptomatic | -- | 114 (87.7%) |  |
| Mild cognitive impairment | -- | 7 (5.4%) |  |
| Symptomatic | -- | 4 (3.1%) |  |
| No consensus | -- | 5 (3.8%) |  |
| **Amyloid / Tau Classification** |  |  | -- |
| Control | 29 (100%) | -- |  |
| A-/T- | -- | 82 (63.1%) |  |
| A+/T- | -- | 14 (10.8%) |  |
| A+/T+ | -- | 34 (26.2%) |  |
| **Down syndrome type** |  |  |  |
| Full trisomy 21 | **--** | 118 (90.8%) |  |
| Translocation | **--** | 8 (6.2%) |  |
| Mosaicism | **--** | 4 (3.1%) |  |

Abbreviations: APOE = apolipoprotein E; DS = Down syndrome; SD = standard deviation

**Supplemental Table 8.** Mediation Analysis Results

| **GFAP Mediates the Relationship between Amyloid PET and Tau PET** | | | |
| --- | --- | --- | --- |
|  | **Estimate** | **95% Confidence Interval** | **p value** |
| **Average Causal Mediation Effect** | 0.0019 | (0.00007, 0) | *0.038* |
| **Average Direct Effect** | 0.0104 | (0.0074, 0.0100) | *< 0.001* |
| **Total Effect** | 0.0123 | (0.0097, 0.0100) | *< 0.001* |
| **Proportion Mediated** | 15.5% | (6.2%, 36.0%) | *0.038* |

**Supplemental Table 9.** Mediation Analysis Results, Amyloid PET data relies on imaging segmentation via AAL).

| **GFAP Mediates the Relationship between Amyloid PET and Tau PET** | | | |
| --- | --- | --- | --- |
|  | **Estimate** | **95% Confidence Interval** | **p value** |
| **Average Causal Mediation Effect** | 0.0018 | (-0.00009, 0) | *0.048* |
| **Average Direct Effect** | 0.0110 | (0.008, 0.0100) | *< 0.001* |
| **Total Effect** | 0.0128 | (0.010, 0.0100) | *< 0.001* |
| **Proportion Mediated** | 14.3% | (0.47%, 33.0%) | *0.048* |

**Supplemental Table 10.** Mediation Analysis Results

| **GFAP Mediates the Relationship between Amyloid PET and Plasma pTau-217** | | | |
| --- | --- | --- | --- |
|  | **Estimate** | **95% Confidence Interval** | **p value** |
| **Average Causal Mediation Effect** | 0.0037 | (0.0018, 0.0100) | *< 0.001* |
| **Average Direct Effect** | 0.0050 | (0.0035, 0.0100) | *< 0.001* |
| **Total Effect** | 0.0087 | (0.0068, 0.0100) | *< 0.001* |
| **Proportion Mediated** | 42.1% | (23.2%, 59.0%) | *< 0.001* |

**Supplemental Table 11.** Mediation Analysis Results, Amyloid PET data relies on imaging segmentation via AAL

| **GFAP Mediates the Relationship between Amyloid PET and Plasma pTau-217** | | | |
| --- | --- | --- | --- |
|  | **Estimate** | **95% Confidence Interval** | **p value** |
| **Average Causal Mediation Effect** | 0.0035 | (0.0017, 0.0100) | *< 0.001* |
| **Average Direct Effect** | 0.0050 | (0.0034, 0.0100) | *< 0.001* |
| **Total Effect** | 0.0085 | (0.0065, 0.0100) | *< 0.001* |
| **Proportion Mediated** | 41.1% | (22.7%, 59.0%) | *< 0.001* |
